# Impaired Executive Function Mediates the Association between Maternal Pre-Pregnancy Body Mass Index and Child ADHD Symptoms

**DOI:** 10.1371/journal.pone.0037758

**Published:** 2012-06-15

**Authors:** Claudia Buss, Sonja Entringer, Elysia Poggi Davis, Calvin J. Hobel, James M. Swanson, Pathik D. Wadhwa, Curt A. Sandman

**Affiliations:** 1 Departments of Pediatrics, University of California Irvine, School of Medicine, Irvine, California, United States of America; 2 Psychiatry and Human Behavior, University of California Irvine, School of Medicine, Irvine, California, United States of America; 3 Obstetrics and Gynecology, University of California Irvine, School of Medicine, Irvine, California, United States of America; 4 Epidemiology, University of California Irvine, School of Medicine, Irvine, California, United States of America; 5 Department of Obstetrics and Gynecology, Cedars Sinai Medical Center, Los Angeles, California, United States of America; Hôpital Robert Debré, France

## Abstract

**Background:**

Increasing evidence suggests exposure to adverse conditions in intrauterine life may increase the risk of developing attention-deficit/hyperactivity disorder (ADHD) in childhood. High maternal pre-pregnancy body mass index (BMI) has been shown to predict child ADHD symptoms, however the neurocognitive processes underlying this relationship are not known. The aim of the present study was to test the hypothesis that this association is mediated by alterations in child executive function.

**Methodology/Principal Findings:**

A population-based cohort of 174 children (mean age  = 7.3±0.9 (SD) yrs, 55% girls) was evaluated for ADHD symptoms using the Child Behavior Checklist, and for neurocognitive function using the Go/No-go task. This cohort had been followed prospectively from early gestation and birth through infancy and childhood with serial measures of maternal and child prenatal and postnatal factors. Maternal pre-pregnancy BMI was a significant predictor of child ADHD symptoms (F_(1,158)_ = 4.80, p = 0.03) and of child performance on the Go/No-go task (F_(1,157)_ = 8.37, p = 0.004) after controlling for key potential confounding variables. A test of the mediation model revealed that the association between higher maternal pre-pregnancy BMI and child ADHD symptoms was mediated by impaired executive function (inefficient/less attentive processing; Sobel Test: t = 2.39 (±0.002, SEM), p = 0.02).

**Conclusions/Significance:**

To the best of our knowledge this is the first study to report that maternal pre-pregnancy BMI-related alterations in child neurocognitive function may mediate its effects on ADHD risk. The finding is clinically significant and may extrapolate to an approximately 2.8-fold increase in the prevalence of ADHD among children of obese compared to those of non-obese mothers. These results add further evidence to the growing awareness that neurodevelopmental disorders such as ADHD may have their foundations very early in life.

## Introduction

Attention-deficit/hyperactivity disorder (ADHD) is the most common neurobehavioral disorder of childhood. It is estimated to affect between 5 and 10% of all children, with increasing trends over the past decade in diagnosis and treatment [1], [2]. ADHD is a complex disorder with multiple etiologies resulting from the interplay of genetic and environmental risk factors [3]. A growing body of evidence suggests that one etiology of ADHD is exposure to adverse intrauterine conditions that affect brain development [4]. Among the major pre- and perinatal factors that have been associated with ADHD are adverse birth outcomes such as low birth weight and preterm birth and excess exposure during the index pregnancy to maternal smoking, alcohol consumption, gestational diabetes and psychological stress [5], [6], [7], [8], [9], [10], [11], [12], [13], [14]. It has been suggested that an increased inflammatory milieu during gestation represents a biological condition that may be common across and underlie the effects of these disparate risk factors [15]. It is well-established that a fetus developing in an inflammatory milieu is significantly more susceptible to subsequently developing various neurodevelopmental disorders [16], [17], [18]. One particularly potent condition that produces an increased inflammatory milieu during gestation is maternal obesity [19], [20], [21]. Consistent with this line of reasoning, maternal obesity before and during pregnancy has been associated with deficits in neurodevelopmental outcomes during childhood and adulthood, including inattention and hyperactivity [8], [22]. Findings from several [23], [24] but not all studies [25] in independent pregnancy cohorts suggest that maternal pre-pregnancy overweight and obesity is associated with ADHD symptoms in the offspring. A feature common across these previous studies is that child ADHD symptoms have been assessed using subjective teacher and/or parent ratings, and that child neurocognitive deficits that accompany behavioral excesses have not been examined in this context (e.g., impaired executive function, a key neuropsychological deficit manifested in ADHD [26]).

Thus, the aim of the present study was to test the hypothesis that the association between maternal pre-pregnancy BMI and child ADHD symptoms is mediated by deficits in child cognitive function. ADHD symptoms and executive function were assessed in a population-based cohort of children who had been followed prospectively from early gestation. The possible residual confounding effects of maternal characteristics (race/ethnicity, SES, intelligence scores, and depressive symptoms at the time of the child follow-up assessment), pregnancy characteristics (obstetric risk conditions in the index pregnancy, parity, length of gestation, and birth weight percentile), and subsequent child characteristics (BMI percentile, sex and age at assessment) were adjusted statistically or accounted for by study design (exclusionary criteria).

## Results

### Child ADHD Symptoms and Executive Function

Mean severity of rating of ADHD symptoms (as assessed by the CBCL) could range between 0 and 2. The average score in this population-based (i.e., non-clinical) cohort of children was 0.45±0.41 (mean ±SD). No symptoms were reported (score 0) for 19% of the children, the symptom score was between 0 and 1 for 81% of the children, and greater than 1 for 10% of the children. This observed distribution of scores is consistent with expected norms, in that patients diagnosed with ADHD typically have a T score of >60 [27], which corresponds approximately with a CBCL subscale score >1.

The average reaction time on the Go/No-Go task was 539.6±87.3 msec (SD), and the average number of correct trials (out of 50) was 39.8±5.5 (SD). On average, more errors were due to false alarms (response to a No-Go cue, 8.2±4.5 (SD) than to missed trials (no response to a Go cue, 2.1±2.7 (SD). The average performance efficiency (reaction time/number of correct responses) was 13.87±3.3 (SD).

Severity of ADHD symptoms was significantly correlated with performance efficiency on this task (r = 0.36, p<0.001).

### Maternal Pre-pregnancy BMI, Weight Gain During Pregnancy and Child ADHD Symptoms

**Figure 1:**
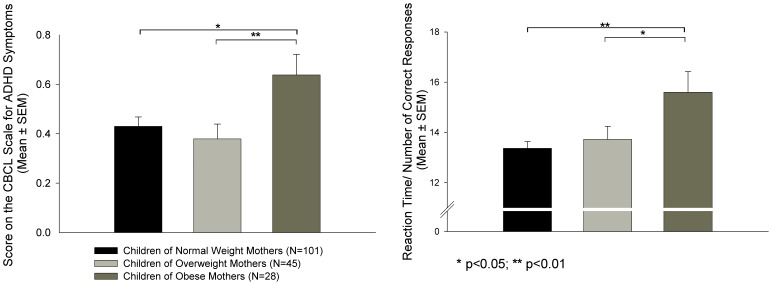
Maternal Pre-pregnancy BMI and Child ADHD Symptoms and Executive Function. Children born to mothers who were obese preconceptionally showed a) higher ADHD symptoms than children born to normal weight or overweight mothers and b) impaired executive function compared to children born to normal weight or overweight mothers. *p<0.05; **p<0.01.

Pre-pregnancy BMI was significantly associated with child ADHD symptoms after controlling for potential confounding variables (F_(1,158)_ = 4.80, p = 0.03). Also, group differences in severity of ADHD symptoms based on pre-pregnancy BMI category were observed (F_(2,157)_ = 4.06, p = 0.02; see Figure 1a). Post-hoc tests revealed that children of obese mothers had significantly greater symptom severity than children of normal weight (p = 0.02) or overweight (p = 0.008) mothers. No difference in ratings of ADHD symptoms was observed between offspring of normal weight and overweight mothers (p = 0.48). On average, there was a 0.22 difference in severity of ADHD symptoms between children from obese pregnancies vs. children from overweight or normal weight mothers (unadjusted effect; Cohen’s *d* effect size  = 0.54 standard deviation (SD) units). Six out of 28 (21.4%) children of obese mothers had a CBCL score >1 (which corresponds to the cut-off for a diagnosis of ADHD [27], compared to 11 out of 146 (7.5%) children of normal weight or overweight mothers (Chi-Square: 5.15, p = 0.02).

Maternal gestational weight gain was not significantly associated with child ADHD symptoms (F_(1,158)_ = 0.03, p = 0.86).

### Maternal Pre-pregnancy BMI, Weight Gain During Pregnancy and Child Executive Function

Maternal pre-pregnancy BMI was significantly associated with child performance on the Go/No-go task (F_(1,157)_ = 8.37, p = 0.004). Also, significant differences in child performance on the Go/No-go task were observed based on BMI category (F_(2,156)_ = 3.57, p = 0.03; see Figure 1b). Children of obese mothers had higher scores for the measure of inverse efficiency, indicating less efficient processing than children born to normal weight (p = 0.002) and overweight (p = 0.02) mothers. Children of overweight mothers did not differ in performance on this task from offspring of normal weight mothers (p = 0.90). On average, there was a 2.06 difference in the measure of performance efficiency (ratio of mean reaction time to the number of correct responses) between children from obese pregnancies vs. children from overweight or normal weight mothers (unadjusted effect; Cohen’s *d* effect size  = 0.62 standard deviation (SD) units).

Additional analyses on mean reaction time and types of errors were performed to explore whether the less efficient task performance was due to impulsive behavior or inattention. Maternal pre-pregnancy BMI was not associated with reaction time (BMI continuous: F_(1,157)_ = 2.46, p = 0.12, BMI categorical: F_(2,156)_ = 0.62, p = 0.54) or number of false alarms (pressing the button on No-go trials, BMI continuous: F_(1,157)_ = 1.70, p = 0.19, BMI categorical: F_(2,156)_ = 1.91, p = 0.15). However, there was a significant association between pre-pregnancy BMI and a higher rate of omission errors (not pressing the button on Go trials, BMI continuous: F_(1,157)_ = 8.38, p = 0.004, BMI categorical: F_(2,156)_ = 3.52, p = 0.03). Post-hoc tests revealed that offspring of obese mothers had a higher rate of omission errors than children born to normal weight (p = 0.002) and overweight (p = 0.02) mothers. Offspring of overweight mothers did not differ in the rate of omission errors from offspring of normal weight mothers (p = 0.58).

Maternal gestational weight gain was not significantly associated with performance on the Go/No-go task (F_(1,157)_ = 0.27, p = 0.61).

**Figure 2:**
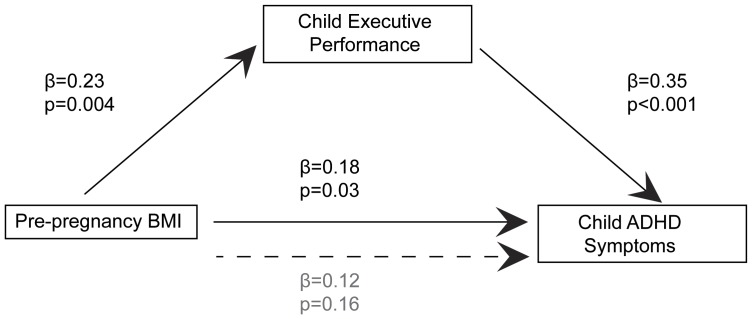
Mediation Analysis for Effects of Maternal Pre-pregnancy BMI on Child ADHD Symptoms via Child Executive Function. The association between pre-pregnancy BMI and severity of child ADHD symptoms was significantly reduced when the measure of performance efficiency in the executive function task was included in the model; Sobel Test: t = 2.39 (±0.002, SEM), p = 0.02.

In the final analytical step we tested whether impaired executive function mediated the association between maternal pre-pregnancy BMI and severity of child ADHD symptoms. This mediation model is summarized in Figure 2. As reported above, maternal pre-pregnancy BMI (independent variable) was significantly associated with child ADHD symptoms (dependent variable, (standardized) β = 0.18, p = 0.03). Furthermore, the association between pre-pregnancy BMI and the mediator (impaired executive function, β = 0.23, p = 0.004), as well as between the mediator and the dependent variable adjusting for the independent variable (β = 0.35, p<0.001), was confirmed. Finally, the association between pre-pregnancy BMI and severity of child ADHD symptoms was significantly reduced when the measure of performance efficiency in the executive function task was included in the model (β = 0.12, p = 0.16, Sobel Test: t = 2.39 (±0.002, SEM), p = 0.02), indicating significant mediation. Thus, as summarized in Figure 2, the association between higher pre-pregnancy BMI and higher severity of ADHD symptoms was partially mediated by impaired executive function (inefficient/less attentive processing).

### Exclusion of Children with Parental ADHD

Exclusion of the six children whose parent or parents had been diagnosed with ADHD from the statistical analyses did not change the significance and the direction any of the above-reported effects.

## Discussion

The current study represents, to the best of our knowledge, the first report demonstrating that the association of maternal pre-pregnancy BMI with subsequent severity of child ADHD symptoms is mediated by alterations in child executive function. Moreover, this effect persists and is substantially unaltered after adjusting for a number of potential confounders, including key pregnancy and birth outcomes and maternal and child characteristics that are established risk factors for ADHD. When examined across clinical categories for BMI, the effect is particularly salient among the children of obese compared to those of overweight or normal pre-pregnancy weight women. The magnitude of the observed effect on child ADHD symptoms and underlying executive function is striking (0.54 and 0.62 standard deviation units, respectively, [28]). In terms of potential clinical significance, these observed differences in ADHD symptoms extrapolate to an approximately 2.8-fold increase in ADHD risk in offspring of obese (21.4% with CBCL score >1) compared to non-obese mothers (7.5% with CBCL score >1).

Our finding that child executive function mediates the association between maternal pre-pregnancy BMI and child ADHD symptoms is important for two reasons. First, it serves to improve our understanding of the underlying etiological mechanism(s). Second, it has clinical implications, in that it suggests a specific, targeted intervention strategy that can be developed and tested in at-risk children of obese mothers. Executive function may be a malleable trait, particularly during critical postnatal developmental periods when key brain networks that sustain attention processing are developing and are maximally plastic [29]. We note that the pattern of performance among the at-risk children in our study of the executive function task (i.e., higher number of omission errors but no difference in reaction time and number of false alarms) is reflective of inattentive rather than impulsive behavior [30]. Specific interventions are now available to train executive function (and particularly attention) in children [31], and it is possible that the application of such interventions may prevent or attenuate subsequent behavioral deficits in this population.

The biological pathways underlying the observed effects in the current study likely relate to inflammation. The state of obesity before and during pregnancy is a major reason for increased systemic inflammation in not only the maternal but also the fetal compartment [20], [21], [32]. Fetal exposure to a pro-inflammatory milieu during critical periods of brain development is believed to represent a key mechanism underlying the development of suboptimal alterations in brain structure and subsequent neurocognitive function. Several experimental studies in animals have shown that exposure to infection or administration of pro-inflammatory cytokines adversely impacts key neurotrophic factors [33], neurogenesis [34], apopotosis [35], neurotransmitter levels [36] and myelination [37]. In fact, a very recent study demonstrated that cell fate itself is altered when neural progenitor cells are exposed to high levels of the pro-inflammatory cytokine interleukin-1β (IL-1β), with gliogenesis being promoted at the expense of neurogenesis [38]. Thus, exposure to infection and high levels of pro-inflammatory cytokines can disrupt the normal developmental trajectory of the fetal brain and produce long-term or permanent consequences for gray matter volume and white matter integrity [17], [37], [39]. It is believed that such alterations in fetal neurodevelopment underlie the association observed in epidemiological studies between exposure to prenatal infection (another condition that is associated with a pro-inflammatory milieu during pregnancy) and increased risk for neuropsychiatric disorders [18]. Biological markers of inflammation were unfortunately unavailable in this study cohort, however, our on-going studies are currently addressing this important issue with a comprehensive characterization of maternal, placental and fetal biology across gestation and birth and serial follow-up assessments in newborns, infants and children using neuroimaging studies of brain structure, white matter maturation and functional connectivity. We also note that fetal exposure to a pro-inflammatory milieu during gestation is a non-specific marker of subsequent ADHD risk, in that it also is associated with a host of other suboptimal mental and physical health outcomes, including child obesity, metabolic dysfunction, and increased risk of other cognitive and affective problems. Thus, it is likely that for any given individual the risk of specifically developing ADHD in the context of inflammation may be influenced (moderated) by additional factors such as genetic vulnerability [40] and other environmental exposures [41].

Consistent with previous reports [23], maternal pre-pregnancy BMI but not gestational weight gain was associated with child ADHD symptoms. This finding mirrors observations related to other outcomes such as child adiposity and insulin sensitivity, wherein maternal pre-pregnancy BMI also is a stronger predictor than maternal weight gain during pregnancy [21]. Furthermore, we recently have reported that pre-pregnancy BMI, not weight or fat gain during pregnancy, is significantly associated with increased C-reactive protein (CRP) levels during pregnancy [42] (one of the best systemic biomarkers of an inflammatory state), suggesting that maternal adiposity before conception may be a stronger determinant of systemic inflammation and its sequelae than gestational weight or fat gain *per se*. This, in turn, raises the intriguing possibility that the state of maternal health prior to pregnancy may have a more important bearing on gestational, birth and subsequent child outcomes than events in the index pregnancy. We note a growing body of evidence is beginning to shed light on the mechanisms by which maternal life-course factors may influence birth and child outcomes [43], [44]. Adopting this life-course perspective to the current findings, it seems reasonable, plausible and even probable that an individual’s susceptibility to developing ADHD may be traced back not only to conditions in intrauterine life but even further back to her or his mother’s state prior to conception. Indeed, pre-pregnancy BMI is a good integrative summary measure of maternal state that captures a host of maternal conditions and exposures over the life course (e.g. nutritional, endocrine and immune/inflammatory state) that have been patterned by her own early programming and pathway mechanisms.

A recent study in two independent cohorts was not able to replicate the association between maternal pre-pregnancy BMI and child attention problems which led the authors to the conclusion that such previously observed associations were due to residual confounding [25]. However, there are notable differences between those studies and the current study. Child behavior was assessed already at 36 and 38 months, respectively, which may be too early to reliably detect attention problems. Furthermore, children of overweight and obese mothers were collapsed in one group and tested against normal weight mothers, which may have diluted the effects of maternal obesity. Thus, while it is possible that the effects of pre-pregnancy BMI may be confounded, exacerbated or attenuated by other pregnancy-related factors and/or postnatal conditions and experiences, important strengths of our study include the prospective, longitudinal design and the ability to address the potential confounding effects of many of these factors by study design (e.g., maternal smoking and drug use). In addition, we assessed and statistically accounted for the possible effects of several other key potential confounding factors (e.g., obstetric risk conditions, maternal depression at the time the CBCL was assessed).

Another strength of the present study is our use of a population-based, as opposed to a clinical, sample of mother-child dyads, in which the distribution of maternal BMI and other maternal and child characteristics closely resembles that of the general population. Therefore, the results of the current study likely are more generalizable than those from studies encompassing or enriched by individuals with a clinical diagnosis of ADHD (which are susceptible to a referral bias).

Obesity during pregnancy is increasing worldwide [45]. Based on recent estimates, about one-third of women in childbearing age in the United States enter their pregnancy obese [19]. Since ADHD confers a major burden on the child, family, society and health care costs, identification of possible modifiable targets for primary intervention is of great interest, importance and relevance. Many questions remain, such as of the precise mechanisms underlying prenatal programming of childhood ADHD by maternal adiposity. To address these questions, a multi-level approach is required that includes molecular and cellular studies, the use of appropriate animal models, and well-designed prospective, longitudinal human studies that incorporate the assessments of immune and metabolic measures during pregnancy [15]. Nonetheless, the results of this study add further evidence to the growing awareness that neurodevelopmental disorders including ADHD may have their foundations in very early life [12], [46].

## Methods

### Participants

Study participants comprised a cohort of children (mean age 7.3±0.9 (SD) yrs, 55% girls) who had been followed prospectively from early gestation and birth through infancy and childhood. Pregnant mothers were recruited in early gestation from the obstetric care provider clinics at the University of California, Irvine, School of Medicine in Orange, CA, and the Cedars Sinai Medical Center in Los Angeles, CA. Exclusionary criteria at the time of recruitment of pregnant mothers included tobacco, alcohol, or other drug use in pregnancy; uterine or cervical abnormalities; or presence of conditions associated with dysregulated neuroendocrine and immune function such as endocrine, hepatic or renal disorders or corticosteroid medication use. Exclusion criteria for children were preterm delivery <34 weeks gestation, perinatal complications associated with neurological consequences (e.g., hypoxia), and congenital, genetic, or neurologic disorders (e.g., fetal alcohol syndrome, Down’s syndrome, fragile × syndrome). Moreover, children of underweight women (pre-pregnancy BMI <18.5) were excluded in the current analysis because maternal under-nutrition also has been shown to alter the offspring’s neurodevelopmental trajectory [47], albeit by a different underlying mechanism than maternal overnutrition. Specifically, while maternal undernutrition could have an impact on the developing brain by, for example, insufficient supply of methyl donors [48] or of critical nutrients like choline [49] that are necessary for normal brain development, the pathways by which maternal obesity and overnutrition affect the developing brain are believed to be related to obesity-associated metabolic and inflammatory alterations [50], [51]. The final sample consisted of 174 mother-child pairs. All methods and procedures included in the current study were approved by the UC Irvine Institutional Review Board and the Cedars-Sinai Medical Center Institutional Review Board. All mothers provided written informed consent, and all children provided informed assent after complete description of the study to the subjects. This consent procedure was approved by the review boards of the participating institutions. The sociodemographic, pre-pregnancy, pregnancy and concurrent maternal and child characteristics of the study population are presented in Table 1.

**Table 1:** Maternal and child characteristics.

Maternal Characteristics	N = 174
***Sociodemographic***	
Race/Ethnicity	
Non-Hispanic White	48% (N = 84)
Hispanic White	19% (N = 33)
Asian	11% (N = 19)
African American	9% (N = 16)
Other	13% (N = 22)
Age at delivery (years, mean±SD)	31.1±5.5
Years of school completed (mean±SD)	15.3±5.5
***Pre-Pregnancy***	
Body mass index (BMI, mean±SD)	25.5±5.9
***Pregnancy***	
Weight gain over gestation (kg, mean±SD)	14.9±5.4
Presence of obstetric risk condition	28% (N = 48)
Primiparous	56% (N = 97)
***Concurrent***	
Maternal intelligence index (percentiles, mean±SD)	50.9±31.0
Concurrent depression score (CESD, mean±SD)	0.32±0.38
**Child Characteristics**	
Sex (females)	55% (N = 95)
Gestational age at birth (weeks, mean±SD)	39.1±1.6
Birth weight percentiles (mean±SD)	56.2±28.0
Age at assessment (mean±SD)	7.3±0.9
BMI percentiles (mean±SD)	67.8±27.3

### Assessments During the Index Pregnancy

#### Maternal anthropometrics

Maternal pre-pregnancy BMI (weight kg/height m^2^) was computed based on pre-pregnancy weight abstracted from medical charts and maternal height measured at the research laboratory during the first visit. Pre-pregnancy BMI was treated both as a continuous and categorical variable. Based on pre-pregnancy BMI, mothers were either classified as normal weight (BMI: 18.5–24.99), overweight (BMI: 25–29.99) or obese (BMI ≥30) in accordance with the Institute of Medicine (2009) recommendations [52].

Maternal weight during pregnancy was measured in the research laboratory up to five times over the course of gestation, at 15 (n = 91), 19 (n = 172), 25 (n = 170), 31 (n = 170) and 37 (n = 142) weeks gestation. Since maternal and fetal weight gain flatten after week 37 [53], [54], hierarchical linear growth curve models [55] were employed to impute estimated weight at 37 weeks gestation for every mother based on available weight measures over the course of gestation. Total gestational weight gain (GWG) was calculated by subtracting the pre-pregnancy weight from the estimated weight at 37 weeks gestation.

#### Obstetric risk, length of gestation and birth weight

Obstetric risk was defined as the presence of major medical complications in the index pregnancy, i.e., gestational diabetes, vaginal bleeding, placenta abruptio, pregnancy-induced hypertension, preeclampsia, or infection. Risk conditions were ascertained by extensive medical chart review and coded as a binary variable (presence or absence of obstetric risk), as previously described [56].

Gestational age was determined by best obstetric estimate with a combination of last menstrual period and early uterine size, and was confirmed by obstetric ultrasonographic biometry before 20 weeks using standard clinical criteria [57].

Birth outcomes were abstracted from medical charts after delivery, and birth weight percentiles were computed using national norms [54].

#### Maternal sociodemographic characteristics

At the first visit during pregnancy, standardized structured interviews were conducted for ascertainment of maternal sociodemographic characteristics (maternal age, education, race/ethnicity).

### Assessments at the Time of Child Follow-up

#### Child Attention-deficit/Hyperactivity Disorder (ADHD) symptoms

Child ADHD symptoms were assessed using the Child Behavior Checklist (CBCL) [58] subscale that reflects attention deficit hyperactivity problems (in accordance with the Diagnostic and Statistical Manual of Mental Disorders-IV [59]). A large number of studies have demonstrated a high concordance between CBCL-based ratings and ADHD diagnosis based on clinical interviews and concluded that it is a reliable screening instrument [58], [60], [61]. The CBCL relies on maternal report, and the ADHD symptom problem behavior subscale consists of 7 statements about the child (“Fails to finish things he/she starts; Can’t concentrate, can’t pay attention for long; Can’t sit still, restless, or hyperactive; Impulsive or acts without thinking; Inattentive or easily distracted; Talks too much; Unusually loud”) for which the mother could choose one of the following responses: “not true (0)”, “somewhat true (1)”, and “very true (2)”. For each child a mean score was computed (which, based on the rating scale, could range between 0 and 2). We refer herein to this ADHD index as “ADHD symptoms.”

#### Child executive function

Child executive function was assessed using an objective Continuous Performance Task (Go/No-go task) that required the execution of an anticipated motor response or its active inhibition. The Go/No-go task is a widely used task for assessment of neurocognitive impairment in ADHD patients [30], [62]. Participants were primed to press a button as quickly as possible in response to the presentation of every letter, except for the letter “X”. In this task there were two blocks of trials. The first block consisted of 25 trials containing 100 percent target stimuli, used to prime participants to respond to the target stimuli. The second block was the response inhibition condition, consisting of 50 trials containing 25 target stimuli (Go trials) and 25 non-target stimuli (No-Go trials). Before commencing with the task, children had to correctly complete five practice trials (three Go trials and two No-Go trials). Overall efficiency of performance on the second block was assessed by a measure combining speed and accuracy by dividing the mean reaction time by the number of correct responses [63]. A higher score indicates poorer performance, which is why this measure is referred to as “inverse efficiency” [64]. Data was not available on this task for one child.

#### Maternal intelligence measures

Mothers were administered the Perceptual Reasoning Index (PRI) of the WAIS-IV [65]. The subscales of this index contribute to the estimate of Performance IQ. This information is important because maternal measures of Performance IQ are associated with offspring neurocognitive function [66] and because maternal obesity and IQ scores are inversely correlated [67] (see Table 1).

#### Maternal depressive symptoms

Maternal depressive symptoms at the time of the child assessment were assessed using the Beck Depression Inventory [68]. Because maternal mood may affect rating of her child on the CBCL [69], all analyses were controlled for concurrent maternal depression.

#### Parental ADHD

A history of either maternal or paternal ADHD was abstracted from available standard family mental health interviews. For six of the children in the study either the mother or father or both had an ADHD diagnosis (for 4 children their mothers; for 3 children their fathers; and for 1 of these children both parents). Five of these children were offspring of normal-weight mothers, and one child was born to a mother who was overweight before she became pregnant. Although these 6 children may carry a greater genetic susceptibility for ADHD, because 5 of these 6 children were born to mothers who were normal pre-pregnancy weight, these cases were not excluded from the study sample, however, all the statistical analyses were repeated excluding these cases to determine whether they altered any of the results.

### Statistical Analyses

Univariate ANOVAs were conducted to examine the associations between maternal pre-pregnancy BMI, maternal gestational weight gain, child ADHD symptoms and child executive function, adjusting for the following potential confounding variables: pregnancy characteristics and birth outcomes (obstetric risk, parity, gestational weight gain, length of gestation and birth weight percentile), child characteristics (child BMI percentile, sex, and age at assessment), and maternal characteristics (race/ethnicity, intelligence scores, education, and concurrent maternal depression). Maternal pre-pregnancy BMI was included in the statistical models as both a continuous variable (to examine effects along the entire distribution) as well as a categorical variable (to distinguish the effects of clinical categories of normal weight *vs* overweight *vs* obese). Post-hoc analyses using the Least Significant Difference (LSD) test were performed to examine the effects of specific BMI group differences.

Next, for statistically significant effects between pre-pregnancy BMI groups, a standardized effect size (Cohen’s *d* statistic) was computed in order to place effects expressed in the original raw units of measurement (i.e., average scores on the CBCL and performance efficiency (ratio of mean reaction time to the number of correct responses)) in the more generic and dimensionless context of standard deviation (SD) units [28].

Finally, regression analyses were performed to test for the potential mediating effect of impaired executive function following the criteria outlined by Barron and Kenny [70]. This entails, first, that there must be a significant relation between the independent variable (maternal pre-pregnancy BMI) and the dependent variable (ratings of child ADHD symptoms). Second, there must be a significant association between the independent variable (maternal pre-pregnancy BMI) and the purported mediator (performance on the Go/No-go task). Third, the mediator must be significantly associated with the dependent variable (ratings of child ADHD symptoms) after controlling for the independent variable (maternal pre-pregnancy BMI). Fourth, the relations between the independent variable and the dependent variable must be significantly reduced when the purported mediator is added to the model (as evaluated by the Sobel test [71]). For each analysis the potential confounding variables listed above were included in the regression models.
